# Multimodal Sensory Stimulation of the Masseter Muscle Reduced Precision but Not Accuracy of Jaw-Opening Movements

**DOI:** 10.3389/fnins.2019.01083

**Published:** 2019-10-10

**Authors:** Birgitta Wiesinger, Birgitta Häggman-Henrikson, Anton Eklund, Anders Wänman, Fredrik Hellström

**Affiliations:** ^1^Department of Odontology, Clinical Oral Physiology, Umeå University, Umeå, Sweden; ^2^Department of Research and Development, Västernorrland County Council, Umeå University, Sundsvall, Sweden; ^3^Department of Orofacial Pain and Jaw Function, Malmö University, Malmö, Sweden; ^4^Centre for Musculoskeletal Research, Gävle University College, Umeå, Sweden

**Keywords:** sensorimotor control, multimodal sensory stimulation, accuracy, precision, jaw movements, head–neck movements, pain, vibration

## Abstract

A functional integration between the trigeminal and craniocervical sensorimotor systems has been demonstrated, with simultaneous jaw and head–neck movements during jaw opening–closing. We previously showed that pain induction in the masseter muscle increased the relative contribution of the neck component of integrated jaw–neck movements. Induced pain or manipulation of proprioception by vibration did not affect accuracy during a jaw-opening task in men. It is not known how multimodal sensory stimulation, with a combination of pain induction and vibration, affects jaw-opening accuracy and precision. The aim was to investigate how jaw–neck movements, and specifically accuracy and precision of jaw-opening, are affected during concomitant nociceptive and proprioceptive stimulation of the masseter muscle. Twenty-one healthy men performed jaw-opening to a target position, defined as 75% of individual maximum jaw opening, during control (Ctr), vibration of masseter muscles (Vib), pain induction in the masseter (Pain), and concomitant vibration and pain induction in the masseter muscle (VibPain). Simultaneous jaw and head movements were recorded with an optoelectronic system and amplitudes calculated for each jaw opening–closing cycle. Accuracy of jaw movements was defined as the achievement of the target position. Precision of jaw movements was defined as the cycle-to-cycle variability from the mean of cycles 2–10 (coefficient of variation, CV). Differences between the trials were analyzed with Friedman’s test, Dunn’s test, and Benjamini–Hochberg correction. There were no significant differences between the trials for jaw movement amplitudes. For head movements, amplitudes for cycles 2–10 were larger during Pain compared to Ctr and Vib (both *p* = 0.034), and larger during VibPain compared to Ctr (*p* = 0.034) and Vib (*p* = 0.035). There were no differences in accuracy of jaw movements between the trials. For precision of jaw movements, the cycle-to-cycle variability was larger during VibPain compared to Ctr (*p* = 0.027) and Vib (*p* = 0.018). For integrated jaw–neck motor strategy, there was a difference between pain and non-pain trials, but no differences between unimodal and multimodal stimulation trials. For achievement of jaw-opening to a target position, the results show no effect on accuracy, but a reduced precision of jaw movements during combined proprioceptive and nociceptive multimodal stimulation.

## Introduction

Jaw function, including jaw opening, biting, and chewing, incorporates functional integration between the jaw and neck sensorimotor system. Thus, in healthy humans, head and jaw movements are coordinated during jaw-opening tasks in both single and rhythmical movements (Eriksson et al., 2000; Zafar et al., 2000). The coordination of movements is characterized by head extension during jaw-opening and head flexion during jaw-closing (Eriksson et al., 1998). These integrated jaw and head movements involve jaw and neck muscles, among others, the masseter, temporal, sternocleidomastoid, and trapezius muscles (Häggman-Henrikson et al., 2013). The simultaneous jaw and head–neck movements during jaw opening–closing tasks (Eriksson et al., 2000; Kohno et al., 2001) have been suggested to be based in a functional relationship between the trigeminal and craniocervical sensorimotor systems (Eriksson et al., 2000), also seen during jaw clenching (Clark et al., 1993), and in the trigeminocervical reflex (Sartucci et al., 1986). It has been proposed that this functional integration between the trigeminal and cervical regions can optimize performance during jaw function, such as jaw opening (Häggman-Henrikson et al., 2006).

We previously demonstrated that experimental pain in healthy individuals can alter jaw–neck motor behavior, underlining the sensorimotor relationship between the jaw and neck regions (Wiesinger et al., 2013). The relationship between trigeminal and craniocervical sensorimotor systems has a neuroanatomical basis, as the subnucleus caudalis in the trigeminal brainstem nuclear complex receives converging sensory inputs from deep and superficial trigeminal, facial, and upper cervical nerves. The subnucleus caudalis is also anatomically overlapping with the upper cervical dorsal horn, since it extends caudally to the upper spinal cord (Sessle et al., 1986). Sensory information from masseter muscle spindles is processed by neurons in the trigeminal mesencephalic nucleus (MesV). These neurons project through synaptic pathways to the trigeminal motor nucleus (Shigenaga et al., 1988), affecting multiple motor neurons including both jaw-opening and jaw-closing muscles (Zhang et al., 2012), as well as the motor nucleus in the cervical spine (Dessem and Luo, 1999). This convergence of various afferent inputs in the subnucleus caudalis may contribute to the regulation of trigeminal motor function (Romaniello et al., 2000).

The study of sensorimotor interactions and coordination has often been conducted by external manipulation of different sensory signals (modalities) and observation of changes in motor control. Common manipulations are experimentally induced pain by injection of hypertonic saline (HS) (Graven-Nielsen et al., 2000), glutamate, or capsaicin (Wang et al., 2010) in humans, or activation of nociceptors by bradykinin (Hellström et al., 2000) or HS (Capra and Ro, 2000) in animals. To manipulate another modality, Loucks and De Nil (2006, 2012) and Wiesinger et al. (2014) used vibration stimulation of proprioceptors, mainly muscle spindles within the masseter muscle. Research with concomitant stimulation of different sensory modalities related to nociception and proprioception in the trigeminal system, using motor control as an outcome, is limited. However, there are studies evaluating motor control outcomes such as balance, postural control, and speed of gait in combination with vibration of neck muscles in patients with neck pain (Beinert et al., 2015; Wannaprom et al., 2018). Vibration affects motor performance (evaluated by balance and speed of gait) differently depending on presence of pain, with decreased motor performance during vibration in healthy individuals and increased performance in patients with neck pain (Wannaprom et al., 2018). Similar results were previously shown for cervical joint position sense, and dynamic and static postural stability in patients with neck pain (Beinert et al., 2015). In the case of integrated jaw and head movements, no studies have investigated the effect of vibration on motor control output in patients with jaw or neck pain or during experimentally induced pain in healthy participants. In our previous study (Wiesinger et al., 2013), where experimental pain was induced by HS injection in the masseter muscle in men, the results showed that the relative contribution of the neck component of the movements increased during pain, indicating an altered strategy for jaw–neck motor control, which is in line with the notion that pain leads to adaptations in motor control with a wide spectrum of adaptation mechanisms, including redistribution of activity within and between muscles, as well as changes at different levels of the motor system (review by Hodges, 2011).

When performing a motor task to a specific target position, the outcome of the task can be assessed by both the accuracy, that is, achievement of the target position, and precision, that is, reproducibility of the performance of the task. In our previous study, nociceptive stimulation by pain induction in the masseter muscle affected the jaw–neck motor strategy, but did not affect the accuracy during a jaw-opening task to a predefined target amplitude (Wiesinger et al., 2013). Also, when manipulating a different sensory modality, proprioception, by vibration of the masseter muscles, neither the accuracy, nor the precision during the jaw-opening task was affected (Wiesinger et al., 2014).

Thus, previous studies suggest a high stability in the jaw–neck motor system during a jaw-opening task when manipulating separate sensory modalities in the masseter muscle in men. It is not known how concomitant multimodal sensory stimulation affects jaw-opening accuracy and precision.

The aim of the present study was to investigate how jaw–neck movements, and specifically accuracy and precision of jaw opening, are affected by concomitant nociceptive and proprioceptive stimulation of the masseter muscle in men.

## Materials and Methods

### Participants

Healthy men were invited to participate in the study. Originally, data for 21 men were collected; out of these, a total of seven were excluded: three due to a necessary change in study design, three due to incorrect application of the vibrator, and one due to a time delay in the start of the movement recording during the pain trial. To retain the statistical power, eight more participants were therefore recruited for a second data collection. Of these, one participant was excluded due to inability to follow given instructions. Thus, the final analysis is based on 21 healthy men aged 20–34 years (SD = 3.9).

A screening questionnaire and a clinical examination of the jaw function were used to determine eligibility for the study. Exclusion criteria were both self-reported – symptoms in the jaw (joint sounds during jaw opening–closing/chewing; pain/tiredness; difficult opening wide; jaw locking); pain in the head, neck, shoulder, or back; ear disease; hearing loss; neurological disorders; impaired balance; diabetes; muscle and joint disease; tumor; body mass index ≥30; elite athletes or persons with very low level of physical activity – as well as established by clinical examination – signs and symptoms of temporomandibular disorder (TMD) according to Research Diagnostic Criteria Axis I (Dworkin and LeResche, 1992). The participants had to abstain from alcohol and analgesics 24 h prior to the experiment. They were informed about the test procedures and that the injection was expected to cause pain of a short duration, but not about the specific aims of the investigation. All participants provided written, informed consent. The study was conducted according to the Declaration of Helsinki. The Regional Ethical Review Board in Umeå approved the investigation.

### Experimental Procedure and Set-Up

The participants performed continuous jaw opening–closing movements from light tooth contact, in the intercuspal position, to a predefined, individual target position, with eyes closed. The target jaw opening position was defined as 75% of the individual maximum jaw opening, as measured with a ruler.

The motor task was performed under four conditions: (1) control (Ctr), (2) vibration of the masseter muscle (Vib), (3) pain induction in the masseter muscle (Pain), and (4) concomitant vibration and pain induction in the masseter muscle (VibPain). The order of Pain and VibPain was randomized in a balanced design, and there was 30 min resting time between the pain injections. The subjects performed one learning trial before the Ctr trial; this was not used in the analysis. The recording time for each trial was 25 s. The four trials were performed in one experimental session. The motor task was performed three times during each trial, only the first recording was used in the analysis.

Participants practiced the individual jaw-opening target before each trial, using a cellular plastic block cut to the individual 75% target position. The target position was registered twice with the optoelectronic recording system (MacReflex^®^, Qualisys), while the subjects held the plastic block between their front teeth. The jaw opening–closing pace was also practiced before each trial, with a metronome set at 50 beats/min. The subjects were instructed to try to maintain a similar speed during the trials, while their main task was to achieve the 75% target position in each jaw opening–closing cycle.

**FIGURE 1:**
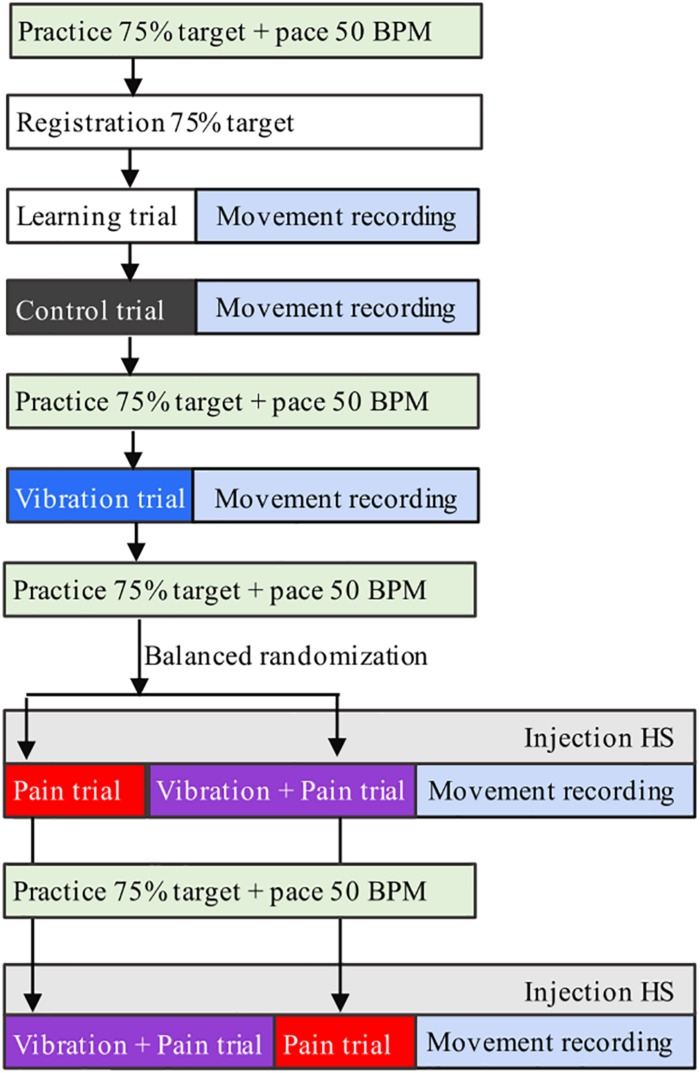
Flowchart of the experimental set-up. Participants (*n* = 21) performed continuous jaw opening–closing movements during four conditions (Control, Pain, Vibration, Vibration, and Pain) in 25 s recordings. HS = 5.8% hypertonic saline.

The participants were seated in an upright position in a chair with back support but without headrest, to allow for free head–neck movements. Before and throughout the experiments, they were given standardized information about the procedure. A flowchart of the experimental set-up is shown in Figure 1.

### Vibration

Bilateral vibration of the masseter muscles was performed with a custom-made device, which has previously been described in detail (Wiesinger et al., 2014). The device was fitted at the start of the session and remained in place until the end. Two plexiglass tubes, attached with an adjustable headband, contained electrical motors and mounted weights, causing vibrations of the tubes. Rubber feet positioned on the bellies of the masseter transmitted the vibrations to the masseter muscles. The application pressure was 2 N on each side, and the vibration frequency was 80 Hz. The vibrators were activated 2 s after the start of movement recording, and the subjects were verbally instructed to start the motor task 1 s after the start of the vibrators.

### Experimental Pain

Pain was induced with unilateral injections of HS (0.2 ml, 5.8%) with a 27G × 3/4^″^ needle into the mid-portion of the masseter muscle over 15 s. The first injection was given on the right side and the second injection, 30 min later, on the left side, regardless of whether the trial included vibration or not (VibPain or Pain). The movement recording started 60 s after the injection of HS, and the subjects started the task 2 s after the start of movement recording.

Participants rated their pain intensity on a 100 mm visual analog scale (VAS), ranging from 0 (no pain) to 100 (worst pain imaginable) 15 and 30 s after the injection, 5 s after the movement recording, and then repeatedly every 15 s up to 4 min 45 s after the injection. Pain rating was not performed during movement recording.

### Concomitant Vibration and Pain Induction

The movement recording started 60 s after injection of HS, the vibrators were activated 2 s after the start of movement recording, and the subjects started the task 1 s after activation of the vibrators.

### Movement Recording

Movements of the jaw and the head were simultaneously recorded in three dimensions with a wireless optoelectronic system (MacReflex^®^, Qualisys) at a sampling rate of 50 Hz. A tripod of retro-reflective markers was attached to the bridge of the nose, and a single marker at the tip of the chin. Two cameras acted as illuminators and detectors of the reflective markers. The set-up enabled movements to be recorded with a spatial resolution of 0.02 mm, within a working volume of 45 cm × 55 cm × 50 cm. Details of the set-up have been described previously (Eriksson et al., 2000; Wiesinger et al., 2013).

### Jaw and Head Movements

**FIGURE 2:**
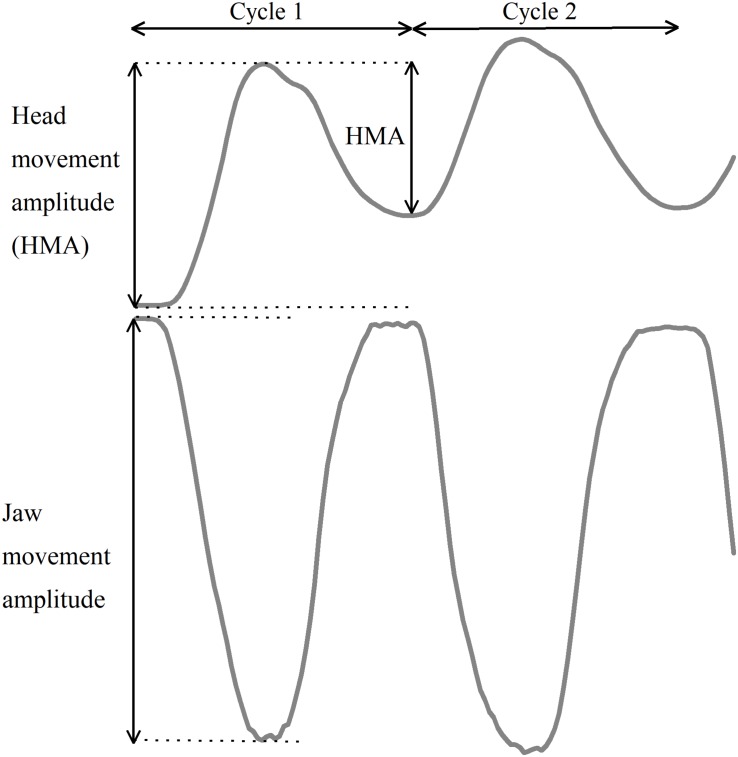
Schematic illustration of the first two jaw opening–closing cycles as well as head and jaw movement amplitudes.

To enable mathematical compensation of the jaw movements for the associated head–neck movements, reference markers were positioned on the head during the recordings. This marker arrangement allowed us to perform a calculation of the jaw movements in relation to the head, thereby compensating for simultaneously occurring head–neck movements. Definitions of head and jaw movement amplitudes and Cycle 1 are defined in Figure 2.

The movement amplitudes for the jaw and head were calculated for each jaw opening–closing cycle. Since the first head movement cycle did not follow the same pattern as the following cycles, the movement amplitude for the first movement cycle was analyzed separately for both jaw and head amplitudes for each individual. The average amplitudes of the jaw and head movements for cycles 2–10 were calculated for each participant in each trial.

The starting point for the jaw movement cycle was defined as the time point at which the mandible began the downward jaw-opening movement. For each movement cycle, the jaw movement amplitude was defined as the distance from the starting position to the most inferior position of the jaw (i.e., at the shift from the jaw-opening phase to the jaw closing phase). For each corresponding movement cycle, the head movement amplitude was defined as the distance between the starting position and the most superior position of the head. The accuracy of the jaw movements was defined as the achievement of the individual 75% target position, and expressed as percentage of target amplitude. The precision of the jaw movements was defined as the intra-individual cycle-to-cycle variability from the mean of Cycles 2–10 of each trial, that is, the coefficient of variation (CV).

### Analysis

Rated pain intensities at specific time points in Pain and VibPain were compared with Wilcoxon matched pairs test. Accuracy of jaw movement amplitudes at different trials was calculated as the percentage of the individual 75% target position. The CV for Cycles 2–10 was calculated for each trial. The overall differences between the trials (Control, Vib, Pain, and VibPain) in Cycle 1 and Cycles 2–10, respectively, were analyzed with Friedman’s test for movement amplitudes, accuracy, and precision. In case of a significant Friedman’s test, *post hoc* comparisons were performed with Dunn’s test. Benjamini–Hochberg correction (Benjamini and Hochberg, 1995) was used to correct the *p*-values for multiple comparisons. Interquartile range was calculated for all variables. The significance level was set at 0.05.

## Results

### Pain Intensity

**FIGURE 3:**
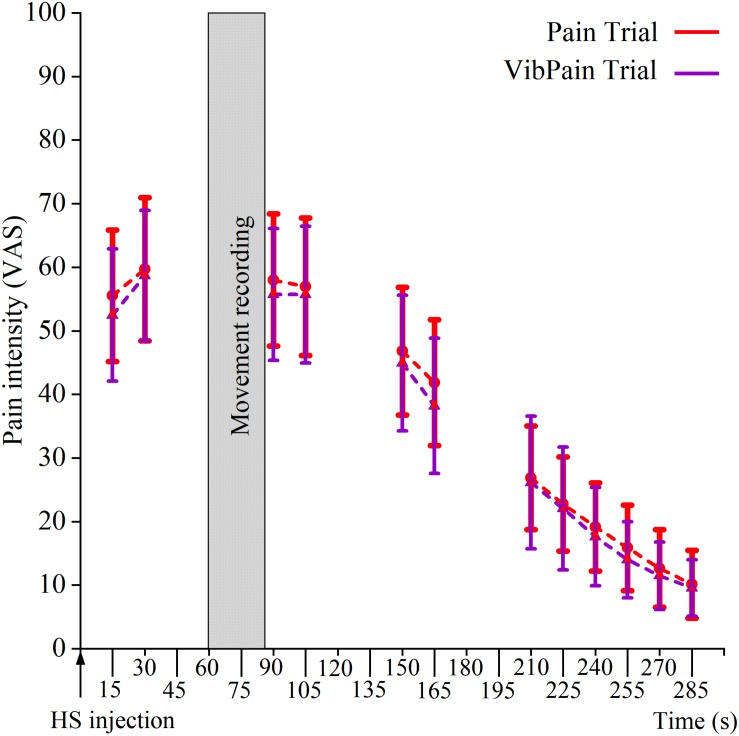
Pain intensity scores (mean and standard error ± 2 SE) on a 0–100 mm visual analog scale following injection of hypertonic saline (HS) in the masseter muscle during Pain (HS injection) and VibPain (concomitant vibration and HS injection) (*n* = 21). Shaded area indicates the time frame of movement recording. *X*-axis shows time (s) after HS injection.

The HS injections initiated local pain in all participants. The pattern for the rated pain intensity was similar for the Pain and VibPain trials with no differences between the trials for specific time points (Figure 3).

### Movement Amplitudes

**FIGURE 4:**
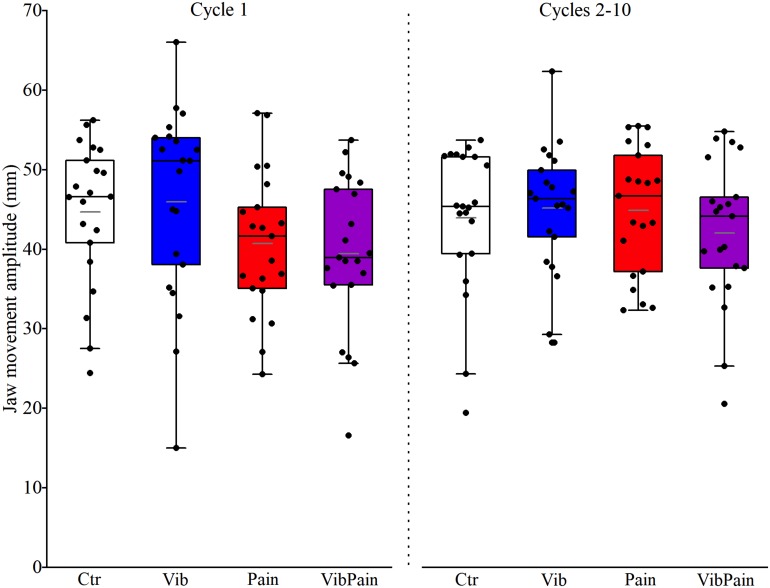
Box plots (median, mean, quartiles, minimum, and maximum) of jaw movement amplitudes during jaw opening–closing tasks in Control (Ctr), Vibration (Vib), Pain, and combined Vibration and Pain (VibPain) trials for the movement cycle 1 and movement cycles 2–10, respectively. Each dot represents one individual.

**FIGURE 5:**
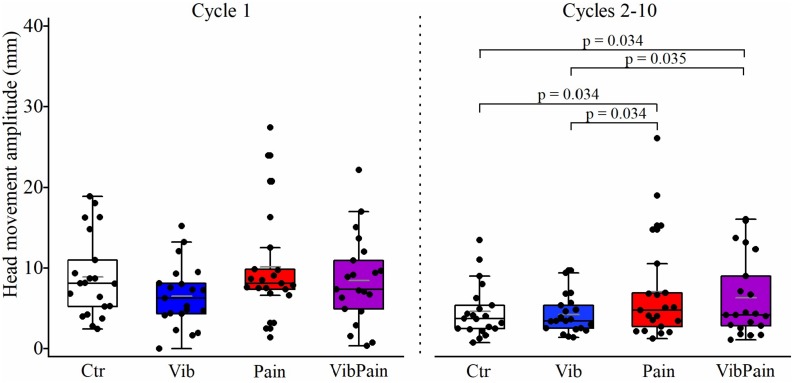
Box plots (median, mean, quartiles, minimum, and maximum) of head movement amplitudes during jaw opening–closing tasks in Control (Ctr), Vibration (Vib), Pain, and combined Vibration and Pain (VibPain) trials for the movement cycle 1 and movement cycles 2–10, respectively. Each dot represents one individual.

There were no significant differences between the different trials for jaw movement amplitudes for Cycle 1 (*p* = 0.074) or Cycles 2–10 (*p* = 0.164) (Figure 4 and Table 1). For head movement amplitudes, there was no overall significant difference between the trials for Cycle 1 (*p* = 0.074), but an overall statistically significant difference for Cycles 2–10 (*p* = 0.009). After corrected Dunn’s tests the head movement amplitudes for Cycles 2–10 were significantly larger during Pain compared to Control and to Vib (both *p* = 0.034), and significantly larger during VibPain compared to Control (*p* = 0.034) and to Vib (*p* = 0.035). There was no difference between Pain and VibPain trials (Figure 5 and Table 1).

**TABLE 1:** Jaw and head movement amplitudes (mm) with interquartile range (IQR) for the different trials.

	**Trial**	**Cycle 1**	**Cycles 2–10**
		**Median (IQR)**	**Median (IQR)**
**Jaw**	Ctr	46.6 (12.2)	45.4 (12.3)
	Vib	51.1 (17.4)	46.4 (10.6)
	Pain	41.7 (11.8)	46.7 (15.5)
	VibPain	39.0 (12.5)	44.2 (12.6)
**Head**	Ctr	8.1 (8.2)	3.7 (3.4)
	Vib	6.2 (4.5)	3.4 (3.1)
	Pain	8.1 (4.1)	4.8 (6.3)^*^
	VibPain	7.4 (6.7)	4.2 (8.0)^*^

*IQR = interquartile range. ^∗^Significant difference from Ctr and Vib after corrected Dunn’s test.*

### Jaw Opening Accuracy and Precision

For accuracy of jaw movements there were no overall differences between the trials for Cycle 1 (*p* = 0.102) or Cycles 2–10 (*p* = 0.164). For precision of jaw movements there was an overall significant difference between the trials (*p* = 0.014). After corrected Dunn’s test the CV was significantly larger during VibPain compared to both Ctr (*p* = 0.027) and to Vib (*p* = 0.018). Table 2 shows the accuracy and precision of jaw movements during the different trials.

**TABLE 2:** Percentage achievement of the individual target position (accuracy) for Cycle 1 and Cycles 2–10, as well as cycle-to-cycle variability (precision) for Cycles 2–10, during the different trials.

	**Accuracy**		**Precision**
**Trial**	**Cycle 1**	**Cycles 2–10**	**Cycles 2–10**
	**% Median (IQR)**	**% Median (IQR)**	**% Median CV (IQR)**
Ctr	95.8 (26.9)	92.9 (21.6)	5.5 (6.0)
Vib	102.5 (21.1)	97.6 (16.7)	5.9 (4.1)
Pain	84.1 (27.9)	97.0 (27.4)	6.4 (3.3)
VibPain	82.8 (32.8)	91.9 (20.4)	7.4(5.9)^*^

*CV = coefficient of variation, IQR = interquartile range. ^∗^Significant difference from Ctr and Vib after corrected Dunn’s test.*

## Discussion

### Main Findings

In this study, integrated jaw and head movements during a jaw-opening task were evaluated during concomitant manipulations of two sensory modalities, proprioception by vibration of the masseter muscle and nociception by HS injections in the masseter muscle. For the integrated jaw–neck motor strategy, there was an overall difference between pain and non-pain trials, but no additional difference between unimodal and multimodal stimulation trials. For the achievement of the jaw opening task to a target position, the results show no overall difference between trials for jaw movement accuracy, but an overall reduced jaw movement precision during multimodal stimulation with vibration and pain induction combined, compared to unimodal stimulation.

### Jaw and Neck Motor Strategy

We have previously shown that experimental pain in healthy individuals altered jaw–neck motor strategy during a jaw-opening task to a target position. The pain-induced change in motor strategy was expressed as an increase of the relative component of the head movements compared to the jaw movements, which remained unchanged (Wiesinger et al., 2013) in both men and women (Wiesinger et al., 2016). This finding, that jaw movement amplitudes remained unchanged during pain induction, despite other reports of reduced jaw amplitudes during experimental pain, was interpreted as an example of task-dependent effects of pain on motor behavior (Sae-Lee et al., 2008). In another study (Wiesinger et al., 2014), we reported that jaw and head movement amplitudes were not affected by vibration of the masseter muscle, indicating a high stability of the jaw–neck motor system. For the combined multimodal stimulation with pain and vibration in the present study, the overall findings were similar to the pain trials, with no change in jaw movement amplitudes, but larger head movement amplitudes compared to the control and vibration trials. This lack of difference in jaw and neck motor strategy, between unimodal and multimodal stimulation trials indicate a general stability of the jaw–neck system. The increase in the relative neck component in pain trials compared to non-pain trials, combined with the stability of jaw movement amplitudes and maintained task achievement, points to goal-oriented performance by the multi joint jaw–neck motor system to ensure the execution of functional jaw tasks.

### Accuracy

In our previous studies of jaw opening to a target position, where, as described above, experimental pain in healthy men altered jaw–neck motor strategy, the accuracy of achieving the jaw-opening target position was not affected (Wiesinger et al., 2013). Neither was the accuracy of jaw opening affected by vibration (Wiesinger et al., 2014). Vibration can affect muscle spindles, sensory organs that convey information on muscle length related to body and limb movement and position movements, as well as other rapidly adapting afferents (Goodwin et al., 1972; Roll et al., 1989). A human study concluded that vibrations with frequencies of 80–100 Hz activated the largest number of muscle spindle endings (Roll et al., 1989). It has previously been reported that vibration can decrease the accuracy of target movements in both extra-trigeminal (Inglis and Frank, 1990) and trigeminal areas (Loucks and De Nil, 2006, 2012), presumably by increased firing of muscle spindles causing an illusion of larger muscle extension than actually performed. In terms of achievement of the target position in our previous study, an undershoot was seen in the control trial, but only for women in the pain trial, and with large individual variations (Wiesinger et al., 2016). Our finding in the present study that vibration did not affect the undershoot meant that we could therefore not confirm previous reports of increased undershoot when vibrating executing muscles. The experimental set-up and the vibration device used in our experiments have also been utilized in another study, where an effect on fine motor control was demonstrated during a bite–hold task (Kumar et al., 2019), indicating that our experimental set-up does affect the proprioception. Taken together, the results from the above studies indicate that for vibration, as for experimental pain (Sae-Lee et al., 2008), the effects, or lack thereof, on jaw motor behavior may be task dependent. Furthermore, the results indicate an overriding stability of the jaw system when performing a goal-orientated task, jaw-opening to a target position, during unimodal stimulation with experimental pain and vibration, as well as multimodal stimulation with both pain and vibration.

### Precision

The precision of jaw opening, as evaluated by the cycle-to-cycle variability, was reduced in the multimodal trial with concomitant pain induction and vibration, compared to the control and unimodal vibration trials. Our finding of a reduced precision, in combination with no change in accuracy, is in line with a study by Kumar et al. (2017) where a dissociation between accuracy and precision was found during a biting task, although they reported dynamic changes for both accuracy and precision. Furthermore, the authors reported differences between trigeminal and non-trigeminal systems, where the lack of visual feedback was discussed as a possible contributing factor.

### Multimodal Stimulation With Pain and Vibration

In the present study, a difference in jaw and neck motor strategy between pain and non-pain trials was found, but no additional difference between uni- and multi-modal stimulations. Also, there was no difference in perceived pain intensity between uni- and multi-modal stimulations. This is in contrast to findings in other body regions, where activation of large myelinated fiber by vibration generally produced an analgesic effect (Lundeberg et al., 1988; Kakigi and Shibasaki, 1992; Ward et al., 1996; Weerakkody et al., 2003; Gay et al., 2015).

The analgesic effect produced by activation of large myelinated fibers during vibration is mediated by receptors located in the skin responding to different vibration frequencies. In the present study the vibration frequency was 80 Hz. It has been suggested that the Pacinian channels should be responsible for the analgesic effect in stimulation ranges from 60 to 400 Hz (Pertovaara, 1979). This could explain the lack of analgesic effect seen in this study, since the skin in the orofacial area lacks functional Pacinian channels (Barlow, 1987; Hollins et al., 1991) or demonstrated afferents from Pacinian corpuscles (Johansson et al., 1988). However, spontaneous TMD pain is shown to be reduced by 100 Hz vibration applied in the orofacial area; thus, the Pacinian corpuscles cannot be essential for the analgesic effect, and other receptors could be involved, for instance, the Meissner’s corpuscles (Roy et al., 2003). The Meissner’s corpuscles are mainly activated by frequencies of 20–50 Hz (Gilman, 2002), which is lower than the 80 Hz used in the present study. In the present study, acute pain stimulation by injection of HS in the masseter muscle was used in healthy individuals, as opposed to the study of Roy et al. (2003), where individuals with chronic TMD were used. It cannot be ruled out that the barrage of tactile afferent information evoked by the vibration may have had effects on the central processing of nociceptive information. Chronic TMD is suggested to include alternations in processing of nociceptive information (review by Chichorro et al., 2017); thus, it is not clear if responses to vibration during acute and chronic pain in the orofacial area would be the same.

## Conclusion

For the integrated jaw and neck motor strategy, there was a difference between pain and non-pain trials, but no additional differences between unimodal and multimodal stimulation trials. For the achievement of jaw opening to a target position, the results show no effect on accuracy, but a reduced precision of jaw movements during multimodal stimulation with combined proprioceptive and nociceptive stimulation.

## Data Availability Statement

The datasets generated for this study are available on request to the corresponding author.

## Ethics Statement

This study involving human participants was reviewed and approved by the Regional Ethical Review Board in Umeå. The participants provided their written informed consent to participate in this study.

## Author Contributions

BW, BH-H, AW, and FH contributed to the conception and design of the study. BW, BH-H, and AE conducted the experiments. BW performed the statistical analysis. BW and FH wrote the first draft of the manuscript. All authors contributed to the manuscript revision, read, and approved the submitted version.

## Conflict of Interest

The authors declare that the research was conducted in the absence of any commercial or financial relationships that could be construed as a potential conflict of interest.
